# The multifaceted role of mobile technologies as a strategy to combat COVID-19 pandemic

**DOI:** 10.1017/S0950268820002435

**Published:** 2020-10-13

**Authors:** R. Teixeira, J. Doetsch

**Affiliations:** EPIUnit – Instituto de Saúde Pública, Universidade do Porto, Rua das Taipas, no 135, 4050-600 Porto, Portugal

**Keywords:** Control, COVID-19, mHealth, mobile technologies, pandemics

## Abstract

The coronavirus 2019 (COVID-19) outbreak in China rapidly spread throughout the world, becoming a threatening pandemic with unprecedented consequences. Mobile technologies have led to a revolution in health and their applicability in the context of COVID-19 is promising. In this commentary, we provide an overview of the role that mobile technologies play in the COVID-19 pandemic context and discuss the main issues associated. Four main domains stood out: health communication, prevention, support and research. Strengthening local surveillance systems, geographic contact tracing, support for clinical practice and data collection of real-time longitudinal data at the population level are some of the main advantages of the applications reported so far. The potential conflict to data privacy urges for discussion on their use in a responsible manner. Along with fair regulation and close monitoring of data collection and process, data anonymisation must be a standard and personal data must be deleted after its usage. Preparation is key for effective containment of a public health crisis and learning lessons on the role of mobile technologies is useful for future challenges in global health. It is noteworthy that their use must be driven by an equitable and inclusive orientation, and mostly integrated into an articulated policy to respond to the crisis.

## COVID-19: a pandemic provoking the need of new approaches

The global dissemination of COVID-19 has spread rapidly and has become an alarming reality in all countries worldwide with an unforeseen duration. The pandemic has led to severe collapse of health service capacities and the prolonged incubation period of the virus has hampered the isolation of suspected cases. Rigorous public health measures are widely used to contain the pandemic. Local and international efforts have been mobilised on several fronts, including in the technology sector.

Mobile technologies include wireless fidelity (Wi-Fi), Bluetooth, data networking services for mobile phones, mobile applications and platforms and location-based services such as global positioning system (GPS). Mobile health (mHealth) is defined as the use of mobile devices and technologies to improve the quality of and access to health care [1]. It covers a multitude of countless applications, such as health communication, treatment adherence, recordkeeping, consultation between health care professionals and health surveillance. Novel and constantly modernised mobile technologies have been transforming the health sector. Thus, how can we use mobile technologies to combat and diminish the effects of a pandemic, such as COVID-19?

The role of mobile technologies is multifaceted and the translation of their diversified functionalities into real-world field studies is a reality. These plural functionalities turn into a broad range of solutions to address the needs of all targeted primary users categorised by the Classification of Digital Health Interventions [2]: clients, health care providers, health system managers and data services. Within the context of the pandemic, their high usability allows for information dissemination, fast-paced contact tracing, telemedicine by mobile devices, real-time data collection, convenient monitoring of health status and effective control, which contributes to mitigate and prevent the spread of infections.

## Current evidence: mobile technologies in COVID-19 pandemic

Recently published articles illustrate the importance of the role of mobile technologies for COVID-19. A study, conducted in a paediatric hospital in Geneva, developed a customised mobile app to disseminate up-to-date and validated information about severe acute respiratory syndrome-coronavirus-2 (SARS-CoV-2) to all medical staff. The results suggest that it was an effective and time-saving communication strategy within the institution, increased reassurance among staff in their daily work and reduced misinformation [3].

On surveillance, a study funded by the Health and Medical Research Fund of Hong Kong forecasted the national and global course of the spread of COVID-19 using human mobility data collected through mobile apps and other digital services [4]. Contact tracing via mobile phones is one of the most useful features within this context to identify possible paths of transmission within the community, as it has been demonstrated in previous epidemics such as Ebola [5]. Geographical tracking using geographic information systems (GIS) through mobile devices enables: (i) real-time mapping of cases, (ii) assessing of social media responses to disease spread, (iii) predictive risk mapping using population travel data, (iv) spreaders trajectory tracking and mapping, (v) contacts tracking across space and time [6]. Geographic contact tracking is essential for timely and effective monitoring in response to epidemics.

The usability of cloud technologies has also been exploited to combat the COVID-19 pandemic in China by collecting and integrating multi-source big data, including electronic health records and real-time information about symptoms from a mobile social media application. Beneficial findings on policy making and clinical decision support, prioritisation of resources and follow-up of discharged patients were identified [7]. Mobile applications were also employed to identify and monitor the mental health status as a consequence of the current pandemic situation, both among health professionals and the general public [8].

When it comes to knowledge production, the use of mobile technologies for real-time collection of longitudinal data at population level has the potential to enrich epidemiological research in a fast-paced pandemic environment [9]. In addition, the linkage of this real-time data with ongoing epidemiological studies and biobanks information produces valuable results to build a data-driven response to the pandemic [9]. This resource can help to characterise and quantify the risk, evolution and sequelae of COVID-19 considering factors such as previous health status, regular medication, lifestyle and genetic data.

## Multifacetedness and diversity – a framework conception

**Fig. 1.**
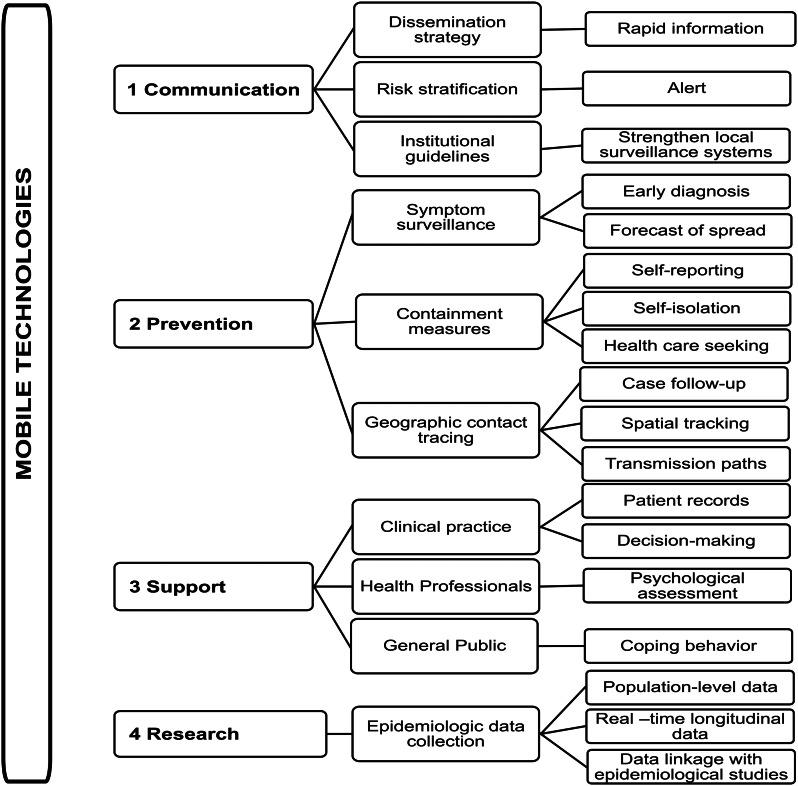
The multifaceted role of mobile technologies.

We developed a framework to illustrate and summarise the multifaceted role of mobile technologies (Fig. 1). Recent findings highlight four main domains where mobile technologies function as an advantageous tool in the COVID-19 pandemic: health communication, prevention, support and research. Firstly, health communication benefits from optimised communication strategies due to high-speed dissemination of information, allowing for risk stratification and alert, and providing easy access to institutional protocols and guidelines. This can assist to strengthen existing local surveillance systems in a cost-effective and time-saving manner. Secondly, mobile technologies favour prevention by convenient symptom surveillance that enable an early detection and forecast of the spread of disease. Mobile technologies facilitate the assessment of containment procedures through self-reporting of health status, self-isolation and early care seeking. Geographic contact tracing enables case follow-up, eases spatial-tracking and early isolation and identifies transmission paths without physical contact. Thirdly, mobile technologies facilitate support for clinical practice and decision making, immediate patient records, mental health, monitoring of front-line healthcare professionals and promotion of coping behaviours for the general population. Lastly, mobile technologies foster epidemiological research through optimised data collection by giving easy access to real-time longitudinal data at a population level, and enabling data linkage with ongoing epidemiological studies.

## Data privacy and protection violation: concerns of mobile technologies

At the same time, despite their enormous potential, innovative technologies implemented during an urgent and accelerated environment of a pandemic raise legal concerns about data protection, privacy and public trust [10]. Apple Inc. and Google recently released the first version of a series of updates to their smartphone operating systems that will use Bluetooth signals from user's phones to alert them if they have been in contact with a person who was tested positive for COVID-19. The ‘Robert Koch Institut’, a German federal government agency and research institute, has recently launched a smartwatch app ‘Corona Datenspende’ (Corona Data Donation). It allows for voluntary and anonymous information sharing through algorithms that recognise symptoms collected by a fitness tracker. In Taiwan, citizens with high risk were quarantined at home and electronically monitored through their mobile phones to guarantee their compliance with quarantine rules during the incubation period [11]. Moreover, immunity certification is in discussion as a key strategy to combat COVID-19 in some European countries. However, evidence is lacking to validate the establishment of long-term or short-term immunity through infection with the virus or a vaccine. Even though mobile or digital technologies might present the safest mean of certifying immunity, they are highly complex and might potentially stimulate controversial political intervention, stigmatisation, abuse and discrimination [12].

## Lawful handling of data: what is the solution?

Despite the predictions of potential benefits in monitoring COVID-19 spread and analyses of the success of containment measures, its intrusiveness into the personal sphere prompts rethinking of data privacy. Massive data collection, along with unlimited and uncontrollable large-scale data storage across multiple servers, calls for attention on this matter. Lawful handling of data considers the consent of a data subject as the adequate basis for data processing according to the European General Data Protection Regulation. In line with the principle of data protection, anonymisation of data must be achieved, either through data that does not relate to an identified or identifiable natural person or data which was altered until non-identifiability.

However, the question remains, whether responsible institutions and professionals in the context of digital surveillance will carry it out appropriately. Furthermore, questions arise, such as whether mobile technologies used for public health to combat COVID-19 will stand above personal choice. Moreover, will the choice become a compulsory action? Authorities in charge should enable and strive for transparency in data processing. Initiatives to deal with privacy concerns related to data location have been surging, such as the use of contact points between individuals to build a network of interactions, dismissing the use of this type of personal data [5]. Another example is Portugal, where a team of researchers from the Institute for Systems and Computers Engineering, Technology and Science (INESC TEC), in collaboration with the Institute of Public Health of the University of Porto (ISPUP), has developed a mobile application called ‘StayAway Covid’ for rapid and, most importantly, anonymous screening of contagion networks by COVID-19. Thus, privacy-by-design must be encouraged by governments. The promotion of an adequate response with fair regulation and close monitoring of the implementation of mobile technologies, preserving citizens' privacy rights is essential. Further, it must be required that mobile apps and platforms delete personal data after they have been used and the crisis has receded.

## Early lessons learned: filling the gaps of tomorrow

In our view, early lessons learned from the role of mobile technologies within the COVID-19 pandemic can prepare us for prospective outbreaks at a minor or major scale. The novel and fast-paced advances in the market of mobile technologies reveal a promising future for challenges in global health. Nonetheless, noteworthy and not to be disregarded are the compliance with data protection, anonymity and confidentiality as the most important key points to focus on. When it comes to precursor conditions, the construction of an enabling environment for the execution of mobile technologies responding to future public health crises, is of equal relevance. Attention must be given to the need of a broad scope of legal structures and ethical frameworks, governance practices, workforce supply, funding requirements and interoperability, i.e. the ability of integrating different information systems.

In the context of public health, mobile technologies will, beyond question, help to set the path for the development and optimisation of strategies to address the multifactorial demand of this pandemic crisis. In such urgent times, harnessing the full potential of mobile technologies to address public health needs is more welcome than ever. However, one cannot overlook important points in this run, such as digital health equity. Policy makers must follow this principle as a transversal guidance to the planning process and implementation of these technologies, considering that digital exclusion is still a prevailing reality in many settings. Ensuring access to all and driving these technologies in ways that reduce health inequalities and benefit underserved populations must be a target.

In addition, we highlight the need of substantial research and evaluation to assess the impact, efficacy and cost-effectiveness in order to optimise public resources utilisation. Current data on the effectiveness and the impact of mHealth solutions in the COVID-19 scene provides yet limited evidence on their ability to improve pandemic response in affected populations. Similarly, there is a paucity of data regarding the clinical impact of mHealth solutions, such as on infection rate, patient's outcome, quality of care and guidelines adherence and dissemination. Therefore, in order to fill these knowledge gaps in the following years, we argue in line with the recommendations of the World Health Organization for digital interventions [2]. Prospective research efforts should examine its effectiveness and resource use, address the compound of gender, equity and rights, and foster collaborations between different digital interventions to establish shared metrics and tools for standardised assessments.

We also strongly recommend strengthening the knowledge exchange among stakeholders to ensure that the developed mHealth solutions meet public health needs. A recent study described the availability of mobile applications addressing the COVID-19 pandemic [13]. Although about 50% of the applications were developed by governments, only 10% were destined for health professionals despite it is already known that this population is at a greater risk of infection than the general population. This unveils a lack of information flow between important key actors, such as health programme managers, researchers and technology developers.

To conclude, we emphasise the fact that mobile technology solutions are not a replacement of an articulated and integrative responsive policy to the pandemic crisis, but rather a supplementary resource to foster these policies.

## Data Availability

This study does not rely on any data, code or other resources.
